# Bioactivity-Guided Fractionation of the Traditional Chinese Medicine Resina Draconis Reveals Loureirin B as a PAI-1 Inhibitor

**DOI:** 10.1155/2017/9425963

**Published:** 2017-09-18

**Authors:** Yu Jiang, Guangping Zhang, Dong Yan, Hong Yang, Zuguang Ye, Tonghui Ma

**Affiliations:** ^1^College of Life Science, Jilin Agricultural University, Changchun, China; ^2^Institute of Chinese Materia Medica, China Academy of Chinese Medical Sciences, Beijing 100700, China; ^3^School of Life Sciences, Liaoning Provincial Key Laboratory of Biotechnology and Drug Discovery, Liaoning Normal University, Dalian 116029, China; ^4^College of Basic Medical Sciences, Dalian Medical University, Dalian, China

## Abstract

Thrombotic diseases have become a global burden due to morbidity, mortality, and disability. Traditional Chinese medicine has been proven effective in removing blood stasis and promoting blood circulation, but the exact mechanisms remain unclear. Plasminogen activator inhibitor-1 (PAI-1) is a natural inhibitor of tissue-type and urokinase-type plasminogen activators. In this study, we screened four fractions of Resina Draconis (a traditional Chinese medicine) extract for PAI-1 inhibitory activity. Bioactivity-guided purification and chromogenic substrate-based assay led to the identification of loureirin B as the major PAI-1 inhibitor, with an IC_50_ value of 26.10 *μ*M. SDS-PAGE analysis showed that formation of the PAI-1/uPA complex was inhibited by loureirin B, and the inhibitory effect of loureirin B on PAI-1 was also confirmed by clot lysis assay. In vivo studies showed that loureirin B significantly prolonged the tail bleeding time and reduced the weight and size of arterial thrombus, reduced hydroxyproline level, and partly cured liver fibrosis in mice. Taken together, the results revealed loureirin B as a PAI-1 inhibitor, adding a new pharmacological target for loureirin B and uncovering a novel mechanism underlying the antithrombotic property of Resina Draconis, which might be useful in the treatment of cardiovascular diseases such as thrombosis and fibrosis.

## 1. Introduction

Thrombotic diseases are caused by the formation of blood clot within the blood vessels, which can obstruct the blood flow through the circulatory system. These diseases may result in disability, morbidity, and mortality, and, therefore, they are considered a global burden. The pathophysiological mechanisms of thrombotic diseases include (1) activation of endogenous and exogenous coagulation system following cardiovascular endothelial cell injury; (2) change in blood status triggered by a decrease in blood flow rate and the formation of blood flow vortex; and (3) high blood solidification caused by an increase in platelets and coagulation factors or a decrease in fibrinolytic activity. Western medicine mainly focuses on the use of antithrombotic drugs such as anticoagulants, platelet aggregation inhibitors, and thrombolytic drugs for treatment. Fibrinolysis is an important process in which fibrin is degraded by plasmin, which is activated by plasminogen activator [1–4], leading to thrombolysis. The fibrinolytic system is composed of plasminogen and plasminogen activators including tissue-type plasminogen activator (tPA), urokinase-type plasminogen activator (uPA), and plasminogen activator inhibitors (PAI). A critical balance of activators and inhibitors is vital to the control of the fibrinolytic system. 

Plasminogen activator inhibitor-1 (PAI-1) is a member of the serine protease inhibitor (serpin) family [5], and it accounts for ~60% of the total PAI in the plasma. As a natural inhibitor of tPA and uPA, PAI-1 acts as a negative regulator in the fibrinolytic system. Thrombotic and fibrotic diseases are largely associated with PAI-1. PAI-1 overexpression can induce spontaneous thrombosis in transgenic mice [6], and PAI-1 knockout protects mice from chemicals and endotoxin [6, 7]. Therefore, inhibition of PAI-1 might be useful in the treatment of such diseases as deep vein thrombosis, coronary syndrome [8], cancer [9], type-II diabetes [10], and venous thromboembolism [11] since PAI-1 is upregulated in these diseases. 

At present, discovering new small molecule PAI-1 inhibitors has become much more pivotal. Several PAI-1 inhibitors have been found, including diketopiperazines XR334, XR330 [12], XR1853, XR5082 [13], and XR5118 and ketopiperazines XR334, XR330 [12], XR1853, XR5082 [13], and XR5118 [14]. Tiplaxtinin (PAI-039) and TM5275 were subsequently identified as the selective PAI-1 inhibitors [7, 10, 15]. Nevertheless, natural compounds are thought to be more beneficial compared to combinatorial compounds in drug discoveries. A variety of traditional Chinese medicines has been used to stimulate blood circulation and remove blood stasis, and this has prompted us to look for PAI-1 inhibitors in these medicinal herbs.

We have previously built a natural product library based on the extracts prepared from 500 commonly used traditional Chinese medicines and found that four of the fractions from the extract of Resina Draconis (also called “dragon's blood”), which is the resin extracted from the stems of* Dracaena cochinchinensis, *have inhibitory effect against PAI-1. According to literatures, dragon's blood has a reputation for facilitating blood circulation and dispersing blood stasis. Pharmacological researches found that dragon's blood has antibacterial, antispasmodic, anti-inflammatory, analgesic, antidiabetic, and antitumor activities, it also could enhance immune function and promote skin repair [16]. The objective of the present study was to identify the active compound(s) from these Resina Draconis fractions and systematically investigate the underlying mechanisms involved in the inhibition of thrombosis and fibrosis.

## 2. Materials and Methods

### 2.1. Chemicals and Animals

PAI-1 Activity Assay Kit, which includes human recombinant PAI-1, uPA, and chromogenic substrates, was purchased from Millipore Corporation (USA). Loureirin B standard was purchased from Tauto (Shanghai) Biotechnology Co., Ltd. Rat hydroxyproline kit was bought from Nanjing Jiancheng Bioengineering Institute. Methanol and acetonitrile were purchased from Honeywell Burdick and Jackson Corporation (USA). Thrombin and other chemicals, unless indicated otherwise, were all obtained from Sangon Biotech (Shanghai) Co., Ltd.

C57BL/6 mice (8–10 weeks) were fed a standard chow diet and kept under specific pathogen-free conditions at Dalian Medical University (Permit Number: SCXK liao 2008-0002).

### 2.2. Isolation and Purification of Loureirin B

Loureirin B was isolated from Resina Draconis using a bioactivity-directed compound isolation strategy. Briefly, Resina Draconis was crushed and extracted with 95% ethanol for 24 h. Fractionation was carried out by preparative HPLC equipped with a constant flow pump (Waters 2525), a dual channel detector (Waters 2487), and an automatic collector (Waters 2767), using a C18 reversed-phase column (XTerra Pre, 5 *μ*m particle size, 19 × 150 mm, USA). The purity of active fractions was checked by an analytical HPLC system (Waters 2695) with a diode assay detector (Waters 2996), using a C18 column reversed-phase column (XTerra OBD, 5 mm particle size, 2.1 × 150 mm, USA), and the structure was determined by comparison with standard.

### 2.3. PAI-1 Activity Assay

Urokinase-type plasminogen activator activity was analyzed using a chromogenic assay as described previously [17]. To evaluate the inhibitory effect of loureirin B on PAI-1 activity, recombinant human PAI-1 was incubated with different concentrations (0, 12.5, 25, 50, 100, 200, and 400 *μ*M) of the compound in a final volume of 100 *μ*L in a 96-well polystyrene plate at 23°C for 15 min. Next, uPA was added to the sample to a final concentration of 0.1 *μ*M followed by incubation at 23°C for 5 min. After that, the samples were transferred to a 37°C incubator for another 15 min, and the proteolytic reaction was initiated by the addition of S-2444 (final concentration 32 *μ*M). The progress of the reaction was monitored by absorbance at 405 nm using a microplate reader. The inhibitory activity of loureirin B against PAI-1 was expressed as percentage relative to the control (no loureirin B).

### 2.4. Clot Lysis Assay

Clot lysis assay was performed based on a previously described method [18], but with slight modification. Briefly, pooled human plasma (5 *μ*L) was dissolved in 65 *μ*L HEPES buffer (150 mM NaCl, 2 mM CaCl_2_, 20 mM HEPES, and pH 7.4) in a 96-well microtiter plate, and fibrin clot formation was initiated by the addition of 10 *μ*L human thrombin (30 NIH IU/*μ*L) followed by 2 h incubation at 37°C. In the meantime, recombinant human PAI-1 (5 *μ*M) was incubated with different concentrations of loureirin B in a 96-well plate at room temperature for 15 min, followed by the addition of uPA (5 *μ*M) and further incubation at 37°C for 5 min. Each of these samples was added to the generated fibrin clots above and the turbidity of the clots was monitored by measuring the absorbance of the samples at 405 nm at every 5 min and over 120 min. All measurements were performed in duplicate. Clot dissolution was expressed as percentage of the control (no loureirin B).

### 2.5. PAI-1/uPA Complex Formation

The effect of loureirin B on the formation of PAI-1/uPA complex was investigated by SDS-PAGE electrophoresis. Loureirin B was diluted with TBS buffer to different concentrations (0, 12.5, 25, 50, and 100 *μ*M) followed by the addition of 10 *μ*L recombinant human PAI-1. After being incubated at 23°C for 15 min, uPA was added to each sample and incubated at 37°C for another 10 min. Next, the samples were heated with nonreduced loading buffer for 3 min and then subjected to SDS-PAGE using 10% Tris glycine gels. Electrophoresis images were obtained using the gel imaging analysis system.

### 2.6. Mouse Arterial Thrombosis Model

The arterial thrombosis model was established according to a procedure described previously [19], but with some modification. Briefly, mice were intraperitoneally given saline or loureirin B at a dose of 1 mg/kg for 2 weeks. For tail bleeding time measurement, the mice were anesthetized with chloral hydrate (50 mg/kg), and the distal segment of the tail (1 cm) was cut off and the rest of the tail was immersed in water with a temperature of 37°C. The bleeding time was recorded. For arterial thrombosis experiment, mice were anesthetized, and the abdomen was cut open to expose the inferior vena artery. A piece of filter paper (1 cm in diameter) saturated with 35% FeCl_3_ was applied to the surface of the vessel for 30 min. Then, the artery thrombus was removed and weighed. Finally, the artery was fixed in formalin, paraffin embedded, sectioned, and stained with hematoxylin-eosin for microscopic observation.

### 2.7. Mouse Liver Fibrosis Model

The effect of loureirin B on liver fibrosis was evaluated by CCl_4_-induced mouse liver fibrosis model. In this experiment, all mice received a regular chow diet. They were orally given 150 *μ*L of olive oil containing 40% CCl_4_ daily over a period of seven weeks and then intraperitoneally injected with saline or loureirin B (1 mg/kg) for the next two weeks. Blood was then collected from the eyeball and the level of serum hydroxyproline in the blood was measured with a hydroxyproline kit. The animals were finally sacrificed by an overdose of anesthetic, and the livers were removed and sliced into thin pieces followed by fixation in formalin. The samples were then observed with a SZX16 microscope (Olympus, Japan).

### 2.8. Statistical Analysis

Data were expressed as means ± SEs or as representative traces. One-way or two-way ANOVA followed by Dunnett's multiple comparison test was used to compare test and control values, and statistical significance was considered at the *P* < 0.05 level.

### 2.9. Ethics Statement

All animal procedures were strictly carried out according to the “Guide for the Care and Use of Laboratory Animals of the National Institutes of Health” and were approved by the Liaoning Normal University Committee on Animal Research. All surgery was performed under chloral hydrate anesthesia, and all possible efforts were made to minimize the suffering of the animals.

## 3. Results

### 3.1. Inhibition of PAI-1 Activity by Resina Draconis and Identification of the Active Component

**Figure 1:**
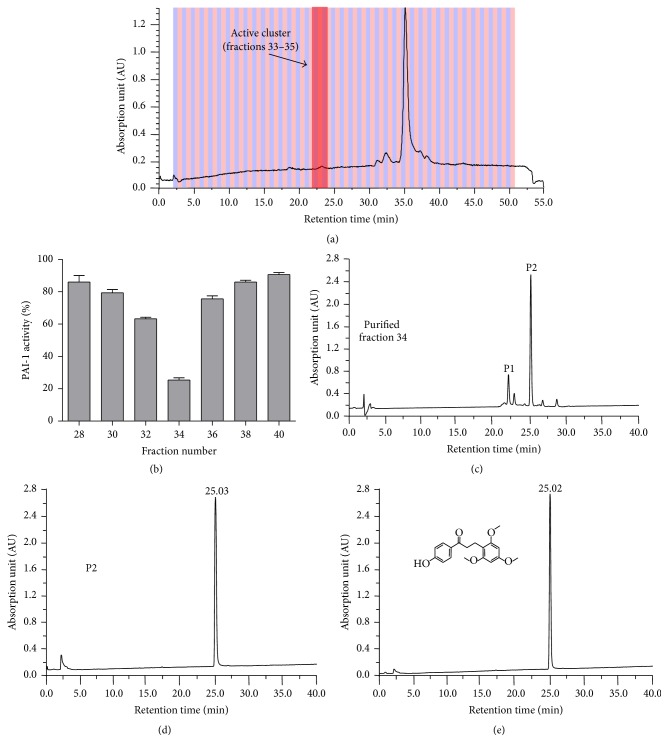
Inhibition of PAI-1 activity by fractions of the Resina Draconis extract and identification of the main PAI-1-inhibiting component. (a) Preparative HPLC chromatogram of the ethanol extract of Resina Draconis. (b) PAI-1 inhibitory activity assay of fractions 28 to 40 of Resina Draconis extract obtained from preparative HPLC. (c) Resolution of fraction 34 of Resina Draconis extract by analytical HPLC. Analytical HPLC chromatogram of P2 of fraction 34 from B (d) and loureirin B standard (e) obtained under the same chromatographic condition. Inset: chemical structure of LB.

Eighty fractions were obtained from the preparation of Resina Draconis extract following preparative HPLC separation. Subsequent activity assay showed that only 3 fractions (33–35) were inhibitory against PAI-I (Figure 1(a)), with fraction 34 inhibiting as much as 75% of the PAI-I activity compared to the control (no Resina Draconis) under the assay condition used (Figure 1(b)). Subsequent resolution of fraction 34 by analytical HPLC yielded a minor peak (P1) and a major peak (P2) (Figure 1(c)). Repeated HPLC run of P2 yielded a purity of more than 98.5% and a retention time of 25.03 min (Figure 1(d)), which corresponded to the retention time of loureirin B (Figure 1(e)), indicating that the purified compound from Resina Draconis was loureirin B.

### 3.2. Characteristic of Loureirin B in the Inhibition of PAI-1

**Figure 2:**
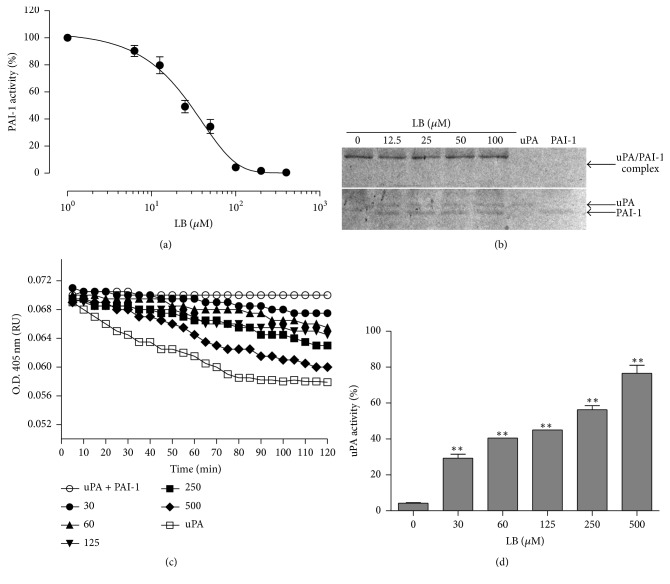
Effect of loureirin B on PAI-1 activity (LB represents loureirin B in the figures). (a) Inhibition of PAI-1 activity by loureirin B. Data are the means ± SEs from four independent experiments. (b) Inhibition of PAI-1/uPA complex formation by loureirin B. PAI-1 and uPA alone or together and in the presence of the indicated concentrations (0–100 *μ*M) of loureirin B. Protein samples were analyzed by SDS-PAGE under nonreducing conditions and visualized by staining with Coomassie brilliant blue. (c) Promotion of uPA-mediated fibrin clot lysis by loureirin B. (d) Recovery of uPA activity by increasing concentrations of loureirin B. Data are the means ± SEs from five independent experiments. “*∗∗*” indicates significant difference at the *P* < 0.01 level.

The inhibitory activity of loureirin B against PAI-1 was tested over various concentrations of loureirin B (Figure 2(a)). The IC_50_ value was determined to be 26.10 ± 2.13 *μ*M. The influence of loureirin B on the PAI-1/uPA complex was investigated using SDS-PAGE. According to the SDS-PAGE result, three bands were detected, and the sizes of these bands were 47 kDa, 57 kDa, and 90 kDa, representing PAI-1, uPA, and PAI-1/uPA complex, respectively (Figure 2(b)). The PAI-1/uPA band was only detected in the absence of loureirin B, and its intensity decreased with increasing concentrations of loureirin B in the sample, suggesting that loureirin B inhibited the formation of uPA/PAI-1 complex, probably through preventing PAI-1 from binding to uPA.

Clot dissolution assay was used to further evaluate the inhibitory effect of loureirin B against PAI-1 activity. PAI-1 was able to inhibit the dissolution of the clot catalyzed by the fibrinolytic activity of uPA, as shown by a lack of reduction in absorbance at 405 nm (Figure 2(c)). However, addition of loureirin B to the sample resulted in progressive increases in clot dissolution even in the presence of PAI-1, clearly showing that the activity of PAI-1 was inhibited by loureirin B, with the inhibition being more intense at higher concentrations of the compound. Increase in cloth dissolution was consistent with the fact that loureirin B relieved the inhibition of uPA exerted by PAI-1 (Figure 2(d)).

### 3.3. Prevention FeCl_3_-Induced Arterial Thrombosis by Loureirin B

**Figure 3:**
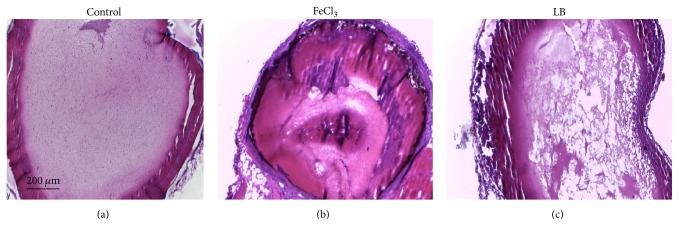
Effect of loureirin B (LB in the figure) on thrombosis induced by FeCl_3_. Control animals were given saline only (a), animals with FeCl_3_-induced thrombosis (b) given no treatment, and animals with FeCl_3_-induced thrombosis given loureirin B as a treatment (c). Mice were injected with LB for two weeks before they were injected with FeCl_3_ to form thrombosis. Only representative picture from each group is shown. Scale bar represents 200 *μ*m.

The in vivo antithrombotic activity of loureirin B was evaluated using FeCl_3_-induced mouse thrombosis model. Compared to the control animals (given saline only), tail bleeding time was prolonged in those given loureirin B, from 11 ± 3.3 min to 15 ± 6.5 min. The thrombus weight of mice given loureirin B was significantly reduced, from 8.8 ± 3 mg to 7.4 ± 3 mg (Table 1). Moreover, loureirin B reduced the wet weight of the arterial thrombus from 8.8 ± 3 mg to 7.4 ± 3 mg. In addition, loureirin B also reduced the area of intravascular thrombosis (Figure 3). The blood distribution and the thickness of blood vessel wall in mice given saline were uniform, with intact and continuous vascular intima structure (Figure 3(a)). On the other hand, the artery of mice treated with FeCl_3_ exhibited uneven intimal lesion, fibrous protein aggregation, and a large area of visible thrombus, and severe damage could be seen in all layers of the vessel wall (Figure 3(b)). Although loureirin B had no effect on the intimal injury, it significantly reduced thrombosis in these mice (Figure 3(c)).

### 3.4. Effect of Loureirin B on CCl_4_-Induced Liver Fibrosis

**Figure 4:**
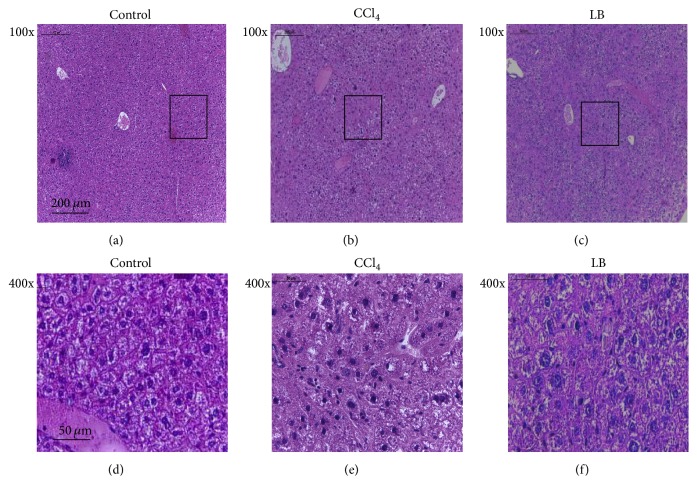
Effect of loureirin B (LB in the figure) on liver fibrosis in mice induced by CCl_4_. (a and d) Control group. (b and e) CCl_4_-treated group. (c and f) CCl_4_-treated group given loureirin B. ((d), (e), and (f)) The magnification of the boxed regions in (a), (b), and (c), respectively.

Overexpression of PAI-1 can lead to liver fibrosis. To investigate the effect of loureirin B on CCl_4_-induced fibrosis formation in mouse liver, we performed histopathological examination measurement. The histology result showed that the hepatic lobule of the control mice was intact, and the hepatic cell cords were well arranged, with no interstitial fiber hyperplasia (Figures 4(a) and 4(d)). Administration of CCl_4_ for seven weeks caused damage to the hepatic lobular structure and resulted in hepatocyte disorder, karyopyknosis, and interstitial fiber hyperplasia (Figures 4(b) and 4(e)). The liver of loureirin B-treated mouse exhibited similar status to that of the control mice, except for the slight fiber hyperplasia (Figures 4(c) and 4(f)). These results suggested that liver fibrosis could be alleviated by loureirin B.

**Figure 5:**
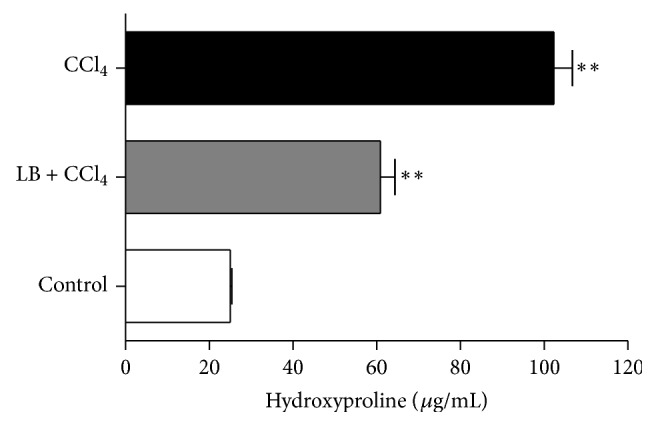
Effect of loureirin B on hydroxyproline content in mice with CCl_4_-induced liver fibrosis. Data are the means ± SEs from six animals. “*∗∗*” indicates significant differences at the *P* < 0.01 level.

Too much collagen and extracellular matrix generation are one of the characteristics of liver fibrosis, and hydroxyproline is a major component of collagen protein. Therefore, measuring the content of hydroxyproline would be a way to assess the extent of liver fibrosis. The content of hydroxyproline in the liver of loureirin B-treated mice was significantly reduced (60.91 ± 0.45 *μ*g/mL) compared to that of the untreated group (102.40 ± 0.42 *μ*g/mL) (Figure 5), suggesting that loureirin B alleviated liver fibrosis in mice.

## 4. Discussion

It is critical to identify active compounds from traditional Chinese herbal medicine with biological activities toward certain molecular targets participating in a particular disease and to understand the therapeutic mechanisms associated with these activities. Resina Draconis is the resin extracted from the stem of* Dracaena cochinchinensis*, and it is commonly called “dragon's blood” in Chinese folk medicine [20]. Resina Draconis has been used to help improve blood circulation since ancient times. It is reported that ethanol extract A of dragon's blood contained pharmacologically effective compounds with antithrombotic effects, partially improving platelet function and anticoagulation activity [21]. However, the exact active components and pharmacological mechanisms of these biological effects have largely remained undetermined. Flavonoids of dragon's blood were later shown to inhibit the formation of deep vein thrombosis in rat [22]. In this study, we showed that Resina Draconis possesses PAI-1 inhibitory activity and isolated and identified the main active compound as the flavonoid loureirin B. Loureirin B inhibited PAI-1 with an IC_50_ of 26.10 *μ*M. It could prevent the formation of uPA/PAI-1 complex and enhance fibrinolysis activity in vitro (Figure 2), properties that could be the basis on which loureirin B reduced the weight of thrombus and extended the bleeding time of FeCl_3_-induced arterial thrombotic mice (Figure 3), thus protecting mice from vascular thrombosis. In addition, loureirin B also weakened liver fibrosis induced in mice by CCl_4_ (Figure 4).

Our study therefore revealed for the first time that loureirin B could be one of the active components responsible for the invigoration of blood circulation by Resina Draconis. Loureirin B has been shown to inhibit hypertrophic scar formation [23, 24], induce Ca^2+^ concentration in rat primary sensory neurons [25], block Kv1.3 channels [26], and modulate sodium current in rat trigeminal ganglion and dorsal root ganglion [27]. The revelation that loureirin B could also act as a PAI-1 inhibitor has not only shed light on its antithrombotic or antifibrotic activity but also revealed a new aspect of its pharmacological activity.

PAI-1 activity can be inhibited by blocking PAI-1/uPA complex formation. There are three forms of PAI-1: active, latent, and substrate [27]. Inactivation of PAI-1 can be achieved by three different mechanisms: (1) preventing the formation of the Michaelis complex; (2) accelerating the transition to the latent conformation; and (3) inducing the turnover of the PAI-1 protease complex as a substrate [27]. In 2012, Xiao et al. identified two PAI-1-inhibiting tanshinone compounds from* Salvia miltiorrhiza* [28], which are also long-known drugs for invigorating blood circulation. The tanshinone compounds exert PAI-1 inhibitory effect through preventing the formation of PAI-1/uPA complex. Formation of the PAI-1/uPA complex and fibrin clot were both decreased with increasing loureirin B concentrations (Figure 2), suggesting that loureirin B, the major component facilitating blood circulation in Resina Draconis, also exhibited inhibitory effect against PAI-1, probably by preventing PAI-1 from binding to uPA, thereby reversing the fibrinolytic activity of uPA. This speculation was consistent with the finding reported by Xiao et al. [28]. Inhibition of PAI-1 might indeed be a common mechanism by which traditional Chinese herbal medicines promote blood circulation.

It is generally accepted that FeCl_3_ can cause oxidative stress and the generation of free radicals, leading to damage of endothelial cells [27], during which PAI-1 level in the platelets rises, causing inhibition of tPA and uPA, and consequently results in the formation of occlusive thrombus [29]. Our data showed that loureirin B prolonged mouse tail bleeding time (Table 1), suggesting that loureirin B may play a positive role in the anticoagulation system through inhibition of PAI-1 level in platelets. Besides, loureirin B also decreased FeCl_3_-induced mouse arterial thrombus weight, demonstrating the protective effect of loureirin B on arterial thrombosis.

Liver fibrosis depends on the generation and degradation of extracellular matrix and collagen which are regulated by the proteolytic activity of uPA/tPA or MMPs. Extracellular matrix and collagen can be degraded by uPA/tPA or MMPs and by acting as an inhibitor of these proteins; PAI-1 also plays a role in this process [30]. Much research has demonstrated that PAI-1 deficiency in mice can lead to increases in tPA and MMP9 activities and decrease in cholestatic liver fibrosis [27] and that PAI-1 level in fibrosis liver is significantly increased [27]. Our data showed that fiber hyperplasia in mice with CCl_4_-induced liver fibrosis was decreased by treatment with loureirin B, resulting in less hydroxyproline accumulation compared to those not treated with loureirin B. This demonstrated that loureirin B was effective at treating liver fibrosis, presumably through inhibiting PAI-1 activity. Additionally, according to Yi et al., six flavonoids have been found in dragon's blood [31], it is possible that there could be other structurally distinct compounds with PAI-1 inhibitory activity, which deserve further investigation.

In conclusion, by isolating and identifying a PAI-1 inhibitor from Resina Draconis, we have effectively revealed a novel mechanism underlying the antithrombotic effect of this traditional Chinese medicine, and one that might explain its therapeutic effect against cardiovascular diseases such as thrombosis and fibrosis.

## Figures and Tables

**Table 1:** Effect of loureirin B on FeCl_3_-induced arterial thrombosis.

Treatment	Bleeding time	Arterial thrombus wet weight
Saline	11 ± 3.3 min	8.8 ± 3 mg
Loureirin B	15 ± 6.5 min^*∗*^	7.4 ± 3 mg

Arterial occlusion evaluated by bleeding time and the arterial thrombus wet weight. “*∗*” represents statistical difference (*P* < 0.05) compared to the control group. Data are the mean ± SEs from six to eight experiments (six for bleeding time experiment, seven for arterial thrombus experiment in saline group, and eight for arterial thrombus experiment in loureirin B group).
